# Prevalence and Risk Factors of *Toxoplasma gondii* Infection among Pregnant Women in Hormozgan Province, South of Iran

**Published:** 2019

**Authors:** Seyedeh Zahra KHADEMI, Fatemeh GHAFFARIFAR, ABDOLHOSSEIN Dalimi, Parivash DAVOODIAN, Amir ABDOLI

**Affiliations:** 1. Department of Parasitology, Faculty of Medical Sciences, Tarbiat Modares University, Tehran, Iran; 2. Department of Biology, Payam Noor University, Tehran, Iran; 3. Infectious and Tropical Disease Research Center, Hormozgan Health Institute, Hormozgan University of Medical Sciences, Bandar Abbas, Iran; 4. Department of Parasitology and Mycology, School of Medicine, Jahrom University of Medical Sciences, Jahrom, Iran; 5. Zoonosis Research Center, Jahrom University of Medical Sciences, Jahrom, Iran

**Keywords:** *Toxoplasma gondii*, Pregnant women, Prevalence, Iran

## Abstract

**Background:**

Toxoplasmosis can cause miscarriage or complications in the fetus. Diagnosis and treatment of this disease by anti-parasitic drugs especially in early pregnancy can help to prevent fetal infection and its complications. This study aimed to determine *T. gondii* infection in pregnant women, evaluate risk factors in the transmission of the disease and congenital toxoplasmosis.

**Methods:**

Overall, 360 sera of pregnant women from 5 cities in the Hormozgan Province in southern Iran with different climate were evaluated from 2015–2016 for *T. gondii* infection by using ELISA method and positive cases of IgM and IgG were tested again using Avidity IgG ELISA. All cases were evaluated according to climate, acute and chronic of toxoplasmosis, number of pregnancy and abortion, epidemiological factors and food habits.

**Results:**

Among 360 specimens *T. gondii* IgG + IgM antibodies were found positive in 0. 8% subjects and also 27% of samples had IgG seropositivity. A significant relationship was observed between age, sampling place, consumption of raw and half cooked meat, history of contact with cats, abortion history, number of children, and parity with IgG positive. In Avidity IgG ELISA test, 13 people with low avidity, 3 people with borderline avidity were reported.

**Conclusion:**

72. 2% of the population had no antibody against the disease that this could be a warning to the people and requires education of preventive and prenatal care and routine screening of women at childbearing age.

## Introduction

Congenital toxoplasmosis is an important infectious cause of abortion and pregnancy complications worldwide (1). Toxoplasmosis is influenced by some factors such as environmental conditions, nutritional and cultural habits, and hygiene. Climate condition can play vital role in oocysts survival. The prevalence of *T. gondii* infection in hot and humid climates is higher than warm and dry areas and is also low in Polar Regions (2).

“Vertical transmission of *T. gondii* to the fetus occurs predominantly in women who acquire the infection at the first time during pregnancy “(3). Congenital toxoplasmosis can lead to a wide variety of manifestations according to gestational age of mothers, while, severe clinical signs are more common in women whose infection was acquired during early gestation (4). In contrast, the risk of vertical transmission is increase with the gestational age, so the highest rates of transmission occur in the last gestation. At this stage, the infection is usually asymptomatic but may develop clinical such as neurological disorders and chorioretinitis at a later age (5). The annual incidence of congenital toxoplasmosis was estimated in the world at 190100 cases. Meticulous information on the “prevalence and risk factors of infection” with *T. gondii* are required to plan proper “prevention measures” against infection during pregnancy and congenital transmission (6).

A number of serological methods have been developed to diagnose *T. gondii* infections (7). However, screening of IgG and IgM antibodies by ELISA method is a routine diagnostic method in clinical laboratories. IgM antibodies appear in the first week of infection and reach at the maximum level after 3 wk of infection. IgG antibodies usually appear two weeks after infection and reach to a peak within 8 to 10 wk of infection. The presence of IgG and absence of IgM indicates the history of previous infection (7,8). Avidity IgG method is particularly useful in detecting new cases as a complementary approach. The primary or immature antibodies have low affinity, but affinity increases with disease proceed and will stay for weeks and months. Avidity index depends on the duration of infection (7).

According to a meta-analysis, the overall seroprevalence rates of *T. gondii* infections is 39. 9% (95% CI: 26. 1–53. 7) among childbearing age women in Iran (9). Moreover, two previous studies in Bandar Abbas (capital of Hormozgan province) showed seroprevalence rates of 49% (10) and 34% (11) in pregnant women. Since there is no comprehensive study on the prevalence of toxoplasmosis in different cities of Hormozgan Province, the present study investigates the prevalence of *T. gondii* infection and related risk factors in a number of pregnant women referring to health centers in Hormozgan Province in southern of Iran. Additionally, IgG avidity test was used as complementary test in the women who were positive for IgG and IgM antibodies to *T. gondii*

## Materials and Methods

### Area of study

Hormozgan Province is located in the north of the Hormuz Strait in southern Iran and cover an area of about 70,697 square kilometers. This province has a very hot and humid climate (ranging between 30–49 °C and humidity of 90%–100% in summers), with an average annual rainfall of 180 mm (12,13).

### Sample size

The study population was pregnant women who referred to the health centers in Hormozgan Province from 2015–2016. The sample size was calculated by the following formula and according to the previous studies in Bandar Abbas that reported the seroprevalence of 38% among pregnant women (10,11). Accordingly, 360 serum samples were collected from pregnant women from 5 different cities of the province (Bandar Abbas, Minab, Haji Abad, Bastak, Qeshm) based on geographic location and climate condition.

$$\text{N}={\text{Z}}^{2}\text{P}\;(1-\text{P})/{\text{d}}^{2}$$

Z=1. 96. P=0.38. d=0.05

### Ethical aspects

The study protocol was approved by the Ethical Committee of Hormozgan University of Medical Sciences Ethical number: (5-HEC-94-3020). All participants were informed about the study, and sampling was conducted with informed consent.

### Sample collection

Two milliliters of blood from women referred to health centers for routine pregnancy tests were collected, and their sera were stored at −20 °C until test. Moreover, information about pregnant women and the risk factors of the infection were gathered by questionnaire at the time of sampling. Questions focused on possible risk factors for infection, including the presence or ownership of animals, eating habits, soil contact, and etc.

### Serological Evaluation

#### Conventional ELISA

The presence of anti-*Toxoplasma* IgM and IgG antibodies were screened using ELISA assay, with an ELISA kit (Pishtaz Teb, Tehran, Iran) according to the manufacturer’s protocol. The positive cut-off value of IgG and IgM antibodies was defined as the upper limit of the 10 and 1. 1 U/mL, respectively.

#### Avidity ELISA

To double check, the results an IgG Avidity test was conducted on 100 samples with the IgM and IgG anti-*Toxoplasma* antibodies using *Toxo* IgG avidity kit (EUROIMMUN, Germany), according to the manufacturer’s protocol. The diagnostic value was defined as relative avidity index (RAI). The avidity index was determined as the following criteria:

RAI <40%: indication of low-avidity antibodies.
RAI 40%–60%: equivocal range.
RAI >60%: indication of high-avidity antibodies.

#### PCR

The final diagnosis of toxoplasmosis in low avidity cases was performed by a 529 bp gene which replicates 200–300 in the *T. gondii* genome (14).

### Statistical analysis

All data were analyzed by SPSS (ver. 20 Chicago, IL, USA) using Chi-square, Cross tab sand Correlate Pearson test.

## Results

Serum samples were collected from 360 pregnant women with a mean age of 27 yr (14–55) who were at different months of pregnancy. Table 1 shows serological findings of pregnant women taken from different cities of Hormozgan province. The percentage of *T. gondii* IgG and IgM+IgG positive antibodies were 27. 8% (100/360) and 0. 83% (3/360) respectively. Three women were seropositive for both IgG and IgM antibodies (Table 1). Table 2 shows history of abortion according to antibody levels. Accordingly, total IgG seropositivity was significantly increased in pregnant women with a history of abortion (*P*=0.01). Risk factors of *T. gondii* seropositivity describe in Table 3. Significant associations were observed between IgG seropositivity and number of pregnancies (*P*=0. 001), age (*P*=0.004), contact to cat (*P*=0.03), consumption of raw or half-cooked meats (*P*=0.05) and raw vegetables consumption (*P*=0.03) (Table 3).

**Table 1:** *T. gondii* serological findings in pregnant women in 5 cities of Hormozgan province, southern Iran

***City***	***Bandar Abbas (N=190) (%)***	***Minab (N=40) (%)***	***Haji Abad (N=40) (%)***	***Bastak (N=40) (%)***	***Qeshm (N=50) (%)***	***Total (%)***
IgG positive	46(24. 2)	10(25)	6(15)	13(32. 5)	25(50)	100(27. 8)
IgM Positive	0(0)	1(2. 5)	1(2. 5)	1(2. 5)	0(0)	3(0. 36)

**Table 2:** Risk factors of *T. gondii* seropositivity among pregnant women in 5 cities in Hormozgan province, southern Iran

***Risk factors***	***Variables***	***N (%)***	***IgG positive N (%)***	***P-value***
Age(yr)	Missing value^†^	24(6. 7)	11(42. 3)	0. 004
	<20	46(12. 8)	10(21. 7)	
	20–39	281(78)	72(25. 6)	
	>40	9(2. 5)	7(77. 7)	
Job	Missing value	22(6. 1)	11(50)	0. 626
	Practitioner	31(8. 6)	7(22. 5)	
	Housekeeper	307(85. 3)	82(26. 7)	
Contact to cat	Missing value	22(6. 1)	11(50)	0. 03
	Yes	26(7. 2)	11(42. 3)	
	No	312(86. 7)	78(25)	
Raw/half-meat	Missing value	26(7. 2)	12(46)	0. 05
	Yes	289(80. 3)	74(25. 6)	
	No	45(12. 5)	14(31)	
Raw vegetables^**^	Missing value	25(7)	12(48)	0. 03
	Yes	12(3. 3)	0(0)	
	No	323(89. 7)	88(27. 2)	
Raw eggs consumption	Missing value	23(6. 4)	11(50)	0. 06
	Yes	95(26. 4)	23(24. 2)	
	No	242(67. 2)	67(27. 7)	

* did not answer.

** Washing with detergent or not.

In avidity IgG method, of 100 seropositive samples, 13 had low avidity

**Table 3:** Comparison of results of IgM ELISA with IgG avidity test in 100 serum samples taken from pregnant women in Hormozgan province

***IgG Avidity***	***IgM ELISA***		***Total N (%)***
	***Positive (%)***	***Negative (%)***	
Low	3(3)	10(10)	13(13)
Borderline	0(0)	3(3)	3(3)
High	0(0)	84(84)	84(84)
Total	3	97	100

*P-*value=0. 6.

### PCR analysis

*T. gondii* DNA was detected in all 13 cases reported low avidity in avidity ELISA method by using 529 base pairs gene.

## Discussion

Toxoplasmosis is an important infection in pregnancy that depending on the time of infection, virulence of the organism type, and number of transmitted parasites from mother to fetus is associated with a variety of symptoms and complications (3).

In this study, *T. gondii* infection rate was 27. 8%. (27% IgG and 0. 83% IgM and IgG). The highest rate (50%) of prevalence was observed in Qeshm Island with hot and humid weather and the lowest prevalence in Haji Abad with hot and dry weather. A significant difference in the infection prevalence was observed in different climates. Probably high temperature weather along with higher humidity causes the sporulated oocyst survival to increase. This increases the probability of the infection prevalence among intermediate and final hosts. In the former studies, scholars reported the similar results for the prevalence, indicating the highest prevalence in the regions with a higher temperate and humidity and the lowest in the hot and arid districts (15). In El Salvador (16), with hot and humid weather, reported that up to 90% of people over 40 yr had anti-*Toxoplasma* antibodies. The results of other surveys show a prevalence of 50% to 80% in different areas of Brazil (17), 15% in China (18), and 22% in London (19).

In Bandar Abbas (provincial capital) the prevalence was 24. 2%. In two similar studies on pregnant women referred to Shariati Hospital in Bandar Abbas (11) 34.2% of women were found with IgG positive and 7.9% of IgM positive and an IgG positive rate of 41. 93%, and IgM positive of zero was reported (10). The reason for the different result of this study compared to the two other studies for Bandar Abbas can come from the population since they selected their samples from the pregnant women referred to Shariati Hospital whereas in the present study in addition to Shariati hospital four other health centers were used to select the sample.

A significant relationship was found between the prevalence of *T. gondii* infection and age confirmed by studies (20) in Turkey and in northern Tehran (21, 22). The findings showed a meaningful relationship between the consumption of raw or half-cooked meats and infection, too. Thus, probably this is one of the ways of infection transmission in the province since the most amount of meat is provided by neighboring provinces such as Fars Province with 37. 5% (23) and Kerman with 24.7% of sheep infection (24). However, so far, no study has been conducted on the livestock of this province. Similar to study (11), a significant relationship was found between the history of contact with cats and the presence of anti-*Toxoplasma* antibodies in pregnant women that indicates another way of transmission in Hormozgan province. In this study, no significant association was observed between toxoplasmosis with jobs and education.

The combination of two sensitive methods inclusive IgM detection and IgG avidity method for *T. gondii* is the best way to determine the time of infection (25). This combination can distinguish between acute and chronic infection (26).

Therefore, all three positive IgM cases had low avidity in avidity IgG method. Moreover, in this study, 10 subjects were negative for IgM but shown low avidity in avidity IgG method. In the follow up for these cases the IgM were positive and the parasite DNA in blood was detectable. Conventional ELISA test cannot often show the new cases of infection corrected by avidity test. A study on the methods of ELISA IgG/IgM and IgG avidity ELISA revealed that IgG avidity is an acceptable approach to distinguish between acute and chronic toxoplasmosis (27). It is a reliable way to screen for the disease, too, especially in the first three months of pregnancy. The high avidity in diagnosis of chronic toxoplasmosis is a valuable index (28). Presence of *T. gondii*-specific IgM antibody in chronic phase of the disease sometimes leads to a wrong diagnosis and may result in an unnecessary abortion. A serum sample with high avidity eliminates the risk of *T. gondii* infection in the first three months of pregnancy (29). The low or borderline avidity antibodies depending on the immune system and the development of IgG antibodies may last for months, so applying the results of avidity test alone can be misleading (30).

Since no correlation was observed between the results of avidity IgG test and levels of IgG and IgM antibodies, both tests should be used for better detection. The presence of high avidity antibodies indicates a definite chronic infection and suggests infection in at least 3–5 months. Therefore, high avidity antibodies are valuable in the first three to four months of pregnancy, but in the following months, there is no diagnostic value (29). Low avidity indicates an acute infection, it cannot be ascertained at a time interval between acute and chronic infection that there is a risk of infection for the fetus. Therefore, this doubt can be resolved by molecular methods.

## Conclusion

In general, screening tests for toxoplasmosis should be performed for pregnant women and for greater certainty, deterministic tests such as avidity IgG are proposed.
